# Invasive Mould Infections in Chronic Granulomatous Disease: A Multicenter Study From Türkiye

**DOI:** 10.1111/myc.70086

**Published:** 2025-07-03

**Authors:** Zeynep Ergenc, Sevgi Bilgic Eltan, Betul Gemici Karaaslan, Ayca Kiykim, Sevgi Aslan Tuncay, Seyhan Yilmaz, Pinar Canizci Erdemli, Aylin Dizi Isik, Burcu Parlak, Mahir Serbes, Adilia Warris, Ahmet Ozen, Elif Karakoc‐Aydiner, Dilek Ozcan, Haluk Cokugras, Safa Baris, Eda Kepenekli

**Affiliations:** ^1^ Pediatric Infectious Diseases Marmara University School of Medicine Istanbul Türkiye; ^2^ Pediatric Infectious Diseases and Immunology Great Ormond Street Hospital for Children London United Kingdom; ^3^ Pediatric Allergy and Immunology Marmara University School of Medicine Istanbul Türkiye; ^4^ The Istanbul Jeffrey Modell Diagnostic Center for Primary Immunodeficiency Diseases Istanbul Türkiye; ^5^ The Isil Berat Barlan Center for Translational Medicine Istanbul Türkiye; ^6^ Pediatric Allergy and Immunology Cerrahpaşa University School of Medicine Istanbul Türkiye; ^7^ Pediatric Allergy and Immunology Çukurova University School of Medicine Adana Türkiye; ^8^ Medical Research Centre for Medical Mycology University of Exeter Exeter United Kingdom; ^9^ Pediatric Infectious Diseases Biruni University School of Medicine Istanbul Türkiye

**Keywords:** *aspergillus*, chronic granulomatous disease, invasive fungal infections, itraconazole, mycoses

## Abstract

**Background:**

Chronic Granulomatous Disease (CGD) is a rare primary immunodeficiency, predisposing to life‐threatening invasive mould infection (IMI). While antifungal prophylaxis has improved outcomes, IMI remains the leading cause of mortality in CGD. This study aimed to evaluate the clinical and fungal epidemiology of IMI among CGD patients in Türkiye and explore diagnostic and treatment challenges.

**Methods:**

Demographics, clinical characteristics, IMI episodes, diagnostic methods, and antifungal prophylaxis regimens of 72 CGD patients followed at the Division of Paediatric Immunology of Marmara, Cerrahpaşa and Çukurova University School of Medicine, Türkiye between 1991 and 2022 were analysed. IMI episodes were classified as proven, probable, or possible based on the European Organisation for Research and Treatment of Cancer/Mycoses Study Group criteria.

**Results:**

Of the patients, 79.1% were male, and 52.8% had autosomal‐recessive CGD (AR‐CGD).

Forty‐two IMI episodes were detected in 39 (54.2%) patients, predominantly involving the lungs. Proven IMI accounted for 28.5% of episodes, with *Aspergillus fumigatus* as the most frequent pathogen. Patients with X‐linked CGD experienced earlier IMI onset than AR‐CGD (34.0 months (IQR: 18.0–65.5) versus 122.0 months (IQR: 40.25–240.0; *p* = 0.005)).

Presentation with IMI led to the CGD diagnosis in 20 (51.3%) patients, while 19 (48.7%) developed IMI under itraconazole prophylaxis (median: 96.0 months, IQR: 48.0–153.0). Of 13 deaths (18.0%), 84.6% were associated with IMI.

**Conclusions:**

Our study highlights the persistently high burden of IMI among CGD patients, despite antifungal prophylaxis. Challenges in diagnosis, including limited access to invasive biopsy and diagnostic modalities, and gaps in prophylactic monitoring, underscore the need for optimised management strategies.

## 1 Introduction

Chronic Granulomatous Disease (CGD), a rare inborn error of immunity, is caused by mutations in the genes encoding the components of the leukocyte nicotinamide adenine dinucleotide phosphate (NADPH) oxidase complex. This enzyme is essential for producing superoxide, which is then converted into hydrogen peroxide and other reactive oxygen species (ROS). These ROS are critical for the intracellular destruction of pathogens by phagocytic leukocytes, such as neutrophils, eosinophils, monocytes, and macrophages [1, 2, 3]. The leukocyte NADPH oxidase complex consists of five subunits. The critical enzymatic component, gp91^phox^ (also known as Nox2), is encoded by the CYBB gene on the X chromosome, and mutations in this gene cause X‐linked CGD (XL‐CGD). Mutations in five autosomal genes lead to autosomal recessive (AR) forms of CGD. These autosomal genes encode the other four subunits: CYBA (encoding p22^phox^), NCF1 (encoding p47^phox^), NCF2 (encoding p67^phox^), NCF4 (encoding p40^phox^) and the recently identified essential chaperone protein for the expression of gp91^phox^, known as cytochrome *b*_558_ chaperone‐1 (CYBC1), encoded by the autosomal gene *CYBC1* [2, 3].

Patients with CGD are highly predisposed to life‐threatening invasive mould infections (IMI) with an incidence of 20%–40% [4, 5, 6]. Despite antifungal prophylaxis and therapy, IMI remains the leading cause of mortality in CGD [6, 7]. *Aspergillus* species are the most frequently isolated moulds, with invasive aspergillosis in this population predominantly attributed to *Aspergillus fumigatus* and *Aspergillus nidulans* [6, 8].

Itraconazole is established as a safe and efficacious agent for primary antifungal prophylaxis in CGD [9, 10, 11]. Posaconazole is a favourable alternative [11, 12]. However, the widespread use of antifungal prophylaxis has contributed to the emergence of azole‐resistant IMI [13, 14]. Consequently, targeted antifungal therapy has become increasingly critical, underscoring the importance of performing invasive diagnostic procedures to accurately identify the causative mould species.

In this study, we aimed to describe the clinical and fungal epidemiology of IMI and the impact of itraconazole prophylaxis in CGD patients in Türkiye.

## 2 Materials and Methods

Patients with CGD followed up in three major referral paediatric immunology centres: Marmara University School of Medicine, Istanbul (follow‐up period: 2007–2022); Cerrahpaşa University School of Medicine, Istanbul (follow‐up period: 1991–2022); and Çukurova University School of Medicine, Adana (follow‐up period: 2003–2022) in Türkiye were included.

Demographic and clinical characteristics of the patients were retrospectively collected from hard copy files and electronic patient records. A diagnosis of CGD was based on either a *DihydroRhodamine* (DHR) and/or *NitroBlue Tetrazolium* (NBT) test. The inheritance pattern was determined through genetic testing and/or a thorough review of the family pedigree. Patient records were reviewed for the presence of IMI, diagnostic test results, antifungal prophylaxis and treatment regimens.

The criteria established by the European Organisation for Research and Treatment of Cancer and the Mycoses Study Group (EORTC/MSG) Education and Research Consortium were utilised for the diagnosis and classification of IMI episodes [15]. A proven IMI episode is defined as one in which a biopsy from a sterile site within the lesion reveals the presence of hyphae through histopathological examination, or when the causative pathogen is identified via culture or polymerase chain reaction (PCR). According to the EORTC/MSG criteria, all patients with CGD inherently fulfil the host factor criteria for classifying an IMI episode as possible when chest imaging reveals compatible findings. Cases are classified as probable when supported by microbiological evidence, such as a positive galactomannan result or positive culture/PCR from a non‐sterile sample, such as bronchoalveolar lavage fluid [15].

Statistical analyses were performed using SPSS Statistics Version 22.0 for Windows (IBM Corp., Armonk, NY). Categorical variables were presented as frequencies and percentages, while continuous variables were expressed as either mean ± standard deviation or median with interquartile range, depending on the normality of their distribution. Continuous variables were compared using the independent‐samples *t*‐test or the Mann–Whitney *U* test based on normality of distribution. The Pearson chi‐square test or Fisher Exact test was used to compare categorical variables, with statistical significance set at *p* < 0.05.

This study was approved by the Local Ethics Committee.

(Reference number 09.2022.607).

## 3 Results

A total of 72 patients with CGD were included. Fifty‐seven (79.2%) were male. Thirty‐eight (52.8%) patients had AR‐CGD, 32 (44.4%) patients had XL‐CGD, and two (2.8%) had undetermined types of inheritance. Mutation type was established in 54 patients: 29 (53.8%) gp91^phox^, 15 (27.8%) p47^phox^, 5 (9.2%) p67^phox^ and 5 (9.2%) p22^phox^. Part of the demographics and clinical characteristics of this study have been previously reported [16].

XL‐CGD patients were diagnosed significantly earlier than AR‐CGD patients with median ages of 19.5 months (interquartile range [IQR] 4.75–48.5) and 114.5 months (IQR 34.25–172.25 months) respectively (*p* < 0.001).

As of 2022, the median age of the 59 surviving patients with CGD, representing 82.0% of the cohort, is 180 months (IQR 112.0–248.0). Among these, 8 patients (13.6%) underwent allogeneic haematopoietic stem cell transplantation (HSCT). The median age of surviving patients with XL‐CGD is 127.0 months (IQR 72.0–187.0), while the median age for those with AR‐CGD is 223.0 months (IQR 165.75–323.0). This difference in median age between XL‐CGD and AR‐CGD patients was statistically significant (*p* = 0.001). The oldest living patient with AR‐CGD and XL‐CGD in the study was 45 years versus 22 years old, respectively, in 2022.

**FIGURE 1:**
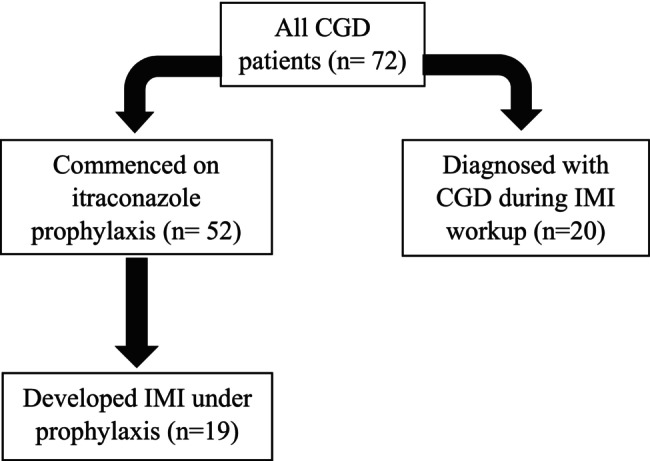
IMI development in CGD patients. CGD, chronic granulomatous disease; IMI, invasive mould infection.

Forty‐two IMI episodes were detected in 39 (54.2%) of 72 patients. Twenty (51.3%) patients were diagnosed with CGD upon presenting with an IMI. Nineteen patients (48.7%) developed an IMI episode under itraconazole prophylaxis for a median duration of 96.0 months (IQR 48.0–153.0) (Figure 1).

XL‐CGD patients experienced significantly earlier first episodes of IMI compared to those with AR‐CGD (*p* = 0.005). The median age of the first IMI in patients with XL‐CGD was 34.0 months (IQR 18.0–65.5), whereas for AR‐CGD, it was 122.0 months (IQR 40.25–240.0). IMI incidence did not significantly differ between XL‐CGD and AR‐CGD (*p* = 0.126). Nevertheless, the percentage of IMI occurrence was higher in the XL‐CGD group (65.6%) compared to the AR‐CGD group (47.3%). No difference was found in the development of IMI within AR‐CGD subtypes (*p* = 0.082). (Table 1).

**TABLE 1:** Characteristics of 72 CGD patients.

	*n* (%)	Male, *n* (%)	IMI, *n* (%)	Presented with IMI, *n* (%)	IMI under itraconazole prophylaxis, *n* (%)	Mortality, *n* (%)	Age of death median (IQR 25–75) (months)
Total	72	57 (79.2)	39 (54.1)	20 (51.2)	19 (48.8)	13 (18.0)	66.0 (19.5‐230.5)
XL‐CGD	32 (44.4)	32 (100)	21 (65.6)	12 (57.1)	9 (42.9)	9 (28.1)	46.0 (19.5‐156.5)
gp91^phox^	29 (40.3)	29 (100)	18 (62.0)	11 (61.1)	7 (38.9)	9 (31.0)	46.0 (19.5‐156.5)
unknown	3 (4.1)	3 (100)	3 (100)	1 (33.3)	2 (66.7)	0	
AR‐CGD	38 (52.8)	25 (65.8)	18 (47.3)	8 (44.4)	10 (55.6)	4 (10.5)	196.0 (26.25‐451.25)
p47^phox^	15 (20.9)	13 (86.7)	4 (26.7)	2 (50.0)	2 (50.0)	1 (6.7)	502.0
p67^phox^	5 (6.9)	3 (60.0)	4 (80.0)	2 (50.0)	2 (50.0)	0	
p22^phox^	5 (6.9)	2 (40.0)	3 (60.0)	0	3 (100.0)	1 (20.0)	93.0
unknown	13 (18.1)	7 (53.8)	7 (53.8)	4 (57.2)	3 (42.8)	2 (15.4)	151.5
Undetermined	2 (2.8)	1 (50.0)	0	0	0	0	

*Note:* Shaded rows indicate the main types of CGD.

Abbreviations: AR, autosomal recessive; CGD, chronic granulomatous disease; IMI, invasive mould infection; XL, X linked recessive.

**FIGURE 2:**
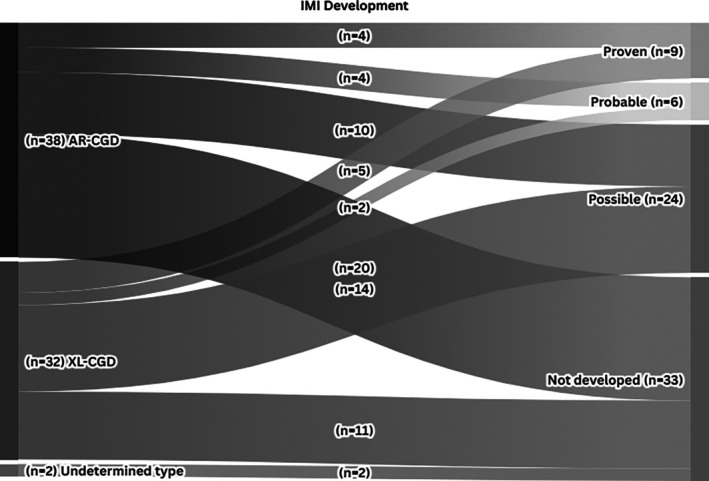
The classification of IMI in CGD patients by inheritance types. AR‐CGD, autosomal recessive chronic granulomatous disease; IMI, invasive mould infection; XL‐CGD, X linked chronic granulomatous disease.

A proven diagnosis of IMI was made in 12 (28.6%) episodes in 9 patients. The remaining episodes were classified as probable in 6 (14.3%) and possible in 24 (57.1%). (Figure 2).

The lungs were the most commonly affected organ, involved in all 42 documented episodes. Bone involvement was identified in four proven episodes and one probable episode, while pleural involvement was reported in three proven episodes. In two proven cases, the lesion extended from the chest wall through the skin.

Among the 9 patients with proven IMI, 5 (55.5%) had XL‐CGD. One XL‐CGD patient experienced three proven episodes, while another with AR‐CGD had two proven episodes. *Aspergillus fumigatus* was identified in five episodes and *Aspergillus flavus* in two episodes. In five episodes, cultures failed to identify the pathogen, but histopathologic studies revealed tissue invasion with hyphae (Table 1). Three patients developed proven IMI while receiving itraconazole prophylaxis. Antifungal susceptibility testing was performed for two of these patients across three episodes, with all isolates found to be sensitive to azoles, including itraconazole.

First‐line treatments included a combination of voriconazole and liposomal amphotericin B for 8 episodes and voriconazole monotherapy for 4 episodes. The median hospitalisation duration was 120 days (IQR 37.0–225.0). Four patients underwent lung surgeries: three lobectomies and one bullectomy. The mortality rate among proven IMI patients was 33.3% (3 out of 9) (Table 2).

**TABLE 2:** Characteristics of the CGD patients with proven IMI episodes.

P/Sex	Inheritance/Mutation	Age at CGD‐diagnosis (months)	Duration of itraconazole prophylaxis before IMI (months)	Age at IMI diagnosis (months)	Involved organs	Chest CT	Histopathology	Culture biopsy/result	IMI outcome
1/M	XL/gp91^phox^	81	113	203	Lungs	Diffuse reticulonodular ground glass opacities with a cavitational consolidation	Septate hyphae with acute angle branching	Lungs/No growth	Died
2/M	XL/gp91^phox^	39	—	39	Lungs, pleura	Bilateral consolidations with pleural thickening, bilateral mediastinal lymphadenopathy, left mediastinal shift with the left lung volume decrease	Septate hyphae with acute angle branching	Pleura/*A.flavus*	Died
3/M	XL/gp91^phox^	26	—	26	Lungs	Bilateral multiple nodular consolidations, mediastinal and subpleural nodules.	Septate hyphae with acute angle branching	Lungs/No growth	Residual changes
4/M	XL/gp91^phox^	15	—	15	Lungs, bone, pleura	Right lung volume decrease, nodular consolidations, right perihilar consolidation with a bronchial compression and invasion in ribs and skin fistula, diffuse pleural thickening.	No suggestive finding	Lungs/*A.fumigatus*	Lobectomy
5/M^a^	XL/gp91^phox^	3	—	2	Lungs, bone, chest wall	Expansive mass across upper mediastinum, bilaterally covering the thoracic inlet, involving the prevertebral, retropharyngeal, retrotracheal, and danger spaces, extending towards the neural foramina and spinal canal at the level of T3‐T4 vertebrae. Anteriorly displaced trachea. Lytic and destructive changes in vertebrae and ribs. Bilateral scattered nodules in lungs and enlarged lymph nodes in the mediastinum	No suggestive finding	Bone/*A.fumigatus*	Bullectomy
6/F	AR/p22^phox^	24	59	83	Lungs, bone	Not available	Septate hyphae with acute angle branching	Bone/*A.flavus*	Died
7/F	AR/p22^phox^	14	20	34	Lungs	Bilateral multiple diffuse reticulonodular densities (the kargest is 6 mm). Bilateral ground glass opacities.	No suggestive finding	Lungs/*A.fumigatus*	Lobectomy
8/F	AR/p67^phox^	126	—	126	Lungs, bone	Multiple nodular consolidation areas on right lung with destruction of ribs. Right mediastinal shift with right lung volume decrease. Multiple nodules on mediastinum and left axilla.	No suggestive finding	Lungs/*A.fumigatus*	Lobectomy
9/M^b^	AR/Unknown	221	—	221	Lungs, pleura	Bilateral multiple nodular consolidations. Pleural thickening. Volume decrease on right lung lower lobe. Traction bronchiectasis.	No suggestive finding	Lungs/*A.fumigatus*	Residual changes

Abbreviations: AR, autosomal recessive; CGD, chronic granulomatous disease; CT, computed tomography; F, female; IMI invasive mould infection; M, male; P, patient; XL, X linked recessive.

^a^ Three proven IMI episodes with *A. fumigatus*.

^b^ Two proven IMI episodes with *A. fumigatus*.

Among the 6 patients with probable IMI, two had positive cultures from bronchoalveolar aspirate samples ( *A. fumigatus* complex, *Aspergillus terreus*) and four others had positive galactomannan tests in addition to chest computed tomography (CT) abnormalities. Four patients were treated with voriconazole monotherapy, while two received a combination of voriconazole and liposomal amphotericin B. The median hospitalisation duration was 45.0 days (IQR 17.0–222.5), with a mortality rate of 50.0% (3/6) (Table 3).

**TABLE 3:** Characteristics of CGD patients with probable IMI episodes.

P/Sex	Inheritance/Mutation	Age at CGD‐diagnosis (months)	Duration of itraconazole prophylaxis before IMI (months)	Age at IMI diagnosis (months)	Involved organs	Chest CT	Histopathology	Serum galactomannan	IMI outcome
10/M	XL/gp91^phox^	10	200	210	Lungs, bone	Bilateral mosaic attenuation, bilateral diffuse reticulonodular densities with traction bronchiectasis. Volume decrease in upper lobes. Consolidation with a rib destruction.	Sputum*/A. fumigatus*	Negative	Died post‐ HSCT
11/M	XL/gp91^phox^	3	—	3	Lungs	Bilateral consolidations with diffuse air bronchograms. Bilateral nodular consolidations	NA	Positive	Died post‐ HSCT
12/M	AR/p67^phox^	11	27	38	Lungs	Bilateral ground glass opacities with nodular consolidations. Lymph nodes in hilus and mediastinum	NA	Positive	Residual changes
13/M	AR/p22^phox^	98	20	118	Lungs	Bilateral ground glass opacities with multiple scattered nodules. Bilateral axillary and mediastinal lymph nodes	BAL/No growth	Positive	Residual changes
14/M	AR/p47^phox^	163	219	502	Lungs	Bilateral ground glass opacities with bilateral multiple scattered nodular consolidations	BAL/ *A. terreus*	Negative	Died
15/F	AR/Unknown	58	26	84	Lungs	Multiple nodules in the right lung	NA	Positive	Residual changes

Abbreviations: AR, autosomal recessive; BAL, bronchoalveolar lavage; CGD, chronic granulomatous disease; CT, computed tomography; F, female; HSCT, hematopoetic stem cell transplant; IMI, invasive mould infection; M, male; NA, not available; P, patient; XL, X linked recessive.

Twenty‐four possible IMI episodes were diagnosed based on radiological findings suggestive of a fungal pneumonia on the chest CT. Ten of these patients were treated with voriconazole monotherapy, four with a combination of voriconazole and liposomal amphotericin B, four with itraconazole, three with posaconazole, and three with liposomal amphotericin B. The median hospitalisation duration was 51.5 days (IQR 23.25–81.75), with a mortality rate of 20.8% (5/24).

Thirty‐two patients (40.6% XL‐CGD) were administered interferon‐gamma (IFN‐γ) therapy, in addition to itraconazole, for varying durations as primary prophylaxis. Of these, 13 (40.6%) patients developed IMI under IFN‐γ therapy. A comparison between patients who received IFN‐γ therapy and those who did not showed no significant difference in IMI incidence (*p* = 0.37).

Allogeneic HSCT was performed in 10 (13.8%) patients, of which 50% had XL‐CGD. Before undergoing HSCT, one patient experienced three proven IMI episodes, three patients had probable IMI episodes, and one patient had a possible IMI episode. After HSCT, two (20%) patients died: one due to sudden infant death and one from a probable IMI occurring post‐HSCT. None of the remaining 8 patients developed IMI during or after HSCT.

In this cohort of CGD with 72 patients, a total of 13 (18.0%) deaths were recorded. Of these, 11 (84.6%) were associated with IMI (3 proven, 3 probable, 5 possible). In 5 of the 11 patients (45.5%), IMI was the presenting symptom of underlying CGD. Among those who succumbed, 7 (53.8%) patients had XL‐CGD, and 4 (30.7%) had AR‐CGD. Median age at death was 66 months (IQR 19.5–230.5).

## 4 Discussion

Our study presents data on the incidence of IMI within a cohort of CGD patients exhibiting a near‐equal distribution of patients with AR‐CGD and XL‐CGD.

In our study 52.8% of CGD cases were AR‐CGD. XL‐CGD has been reported to be more prevalent than AR‐CGD in many places such as Europe, the United States and China [4, 17, 18, 19]. However, AR‐CGD is more prevalent than XL‐CGD in Türkiye likely due to higher consanguinity rates, a pattern also described in Egypt, Iran, Israel and Arab populations [20, 21, 22, 23].

Patients with XL‐CGD are reported to exhibit a higher incidence of IMI compared to those with AR‐CGD, often accompanied by a less favourable prognosis. This disparity is likely attributed to the earlier onset and greater severity of clinical manifestations in XL‐CGD, which facilitate earlier diagnosis and intervention [4, 6, 8, 24]. In our study, however, the incidence of IMI did not significantly differ between patients with XL‐CGD and those with AR‐CGD. Nonetheless, consistent with the existing literature, XL‐CGD patients in our study were diagnosed younger and had IMI episodes earlier in their disease than AR‐CGD patients. Moreover, the median age of surviving patients with XL‐CGD was younger compared to patients with AR‐CGD, further underscoring the poorer prognosis typically associated with XL‐CGD.

Based on the 2020 EORTC/MSG criteria, our retrospective analysis identified 42 IMI episodes in 39 of the 72 CGD patients in our study, with 54.1% experiencing at least one episode. This IMI prevalence is comparable to findings by Beauté et al. who reported that 42.6% of a larger cohort of 155 CGD patients developed at least one invasive fungal disease episode using the earlier 2008 EORTC/MSG criteria [5, 25].

Our study reported a lower proportion of proven IMI compared to previous literature [5, 26]. The lungs are the most commonly affected organ in severe infections associated with CGD, consistent with findings in our cohort [8, 27]. However, the highly invasive nature of lung biopsies, coupled with the substantial risk of complications, limits their routine application in all patients. A study conducted in immunocompromised patients, HSCT recipients, and children with primary immunodeficiencies reported an elevated complication rate of lung biopsies in up to 30% [28]. In our study, 28.5% were classified as proven IMI, whereas Beauté et al. reported higher proportions with 42.5% proven IMI [5]. Henriet et al., in their literature review, reported a notably higher rate of proven invasive fungal disease at 93%, which may reflect an overestimate in published data where proven cases were more likely to be documented [26]. We attribute the lower rate of proven IMI in our study to technical limitations, primarily challenges in performing lung biopsies.

Four CGD patients were classified with probable IMI, based on suggestive chest CT findings alongside a positive serum galactomannan. Nevertheless, it must be acknowledged that the galactomannan test is not validated in CGD and may yield false‐negative results in these patients. Moreover, the diagnostic accuracy of the test may be further diminished by the administration of mould‐active antifungal agents [15, 29].

Nine proven IMI cases were documented in our study. These were caused by *Aspergillus fumigatus* (*n* = 5) and *Aspergillus flavus* (*n* = 2), identified by culture. While cultures did not yield a pathogen in two cases, hyphae were observed in histopathologic studies. During the study period, 18S rRNA fungal PCR and *Aspergillus*‐specific PCR tests were not available. *A. fumigatus* was the most prevalent *Aspergillus* species identified in our study, aligning with previous literature. However, *A. nidulans* , commonly reported as the second most common species in the literature, was not detected in our cohort [5, 6, 8, 29]. Notably, after data collection concluded, *A. nidulans* was identified in an XL‐CGD patient presenting with pulmonary and cerebral involvement.

In our study, 27.8% of patients (*n* = 20) were diagnosed with CGD during an IMI workup; specifically, six were diagnosed after a proven IMI. This observation is consistent with findings from Blumental et al. who similarly reported CGD diagnoses in 5 out of 24 patients after a confirmed IMI episode [29]. These results highlight the critical need to include CGD in the differential diagnosis for patients presenting with IMI.

Itraconazole has long been the preferred antifungal prophylaxis for CGD patients due to its proven efficacy in preventing IMI, favourable safety profile, and low cost [5, 9, 10]. However, more than one‐third (36.5%) of patients in our study developed IMI under primary itraconazole. Beauté et al. reported an even higher incidence of IMI, with half of their patients developing IMI while on itraconazole prophylaxis [5]. This outcome may be attributable to patient non‐adherence, suboptimal exposure (e.g., serum levels), or periodic unavailability of the suspension form of itraconazole in Türkiye [5, 30]. Therapeutic drug monitoring (TDM) of itraconazole is recommended [31]. Unfortunately, TDM for itraconazole was only available for a limited duration during our study, which constrained our ability to evaluate whether our patients achieved therapeutic drug concentrations.

Posaconazole is another agent that has been evaluated for its safety and efficacy to prevent IMI in patients with CGD and is recommended in clinical guidelines for primary antifungal prophylaxis in CGD [11, 12]. Advantages of posaconazole over itraconazole is a better palatability, easier dosing regimen and improved attainment of adequate exposure [32].

The literature presents conflicting findings regarding the clinical value of IFN‐γ in a prophylactic setting. While IFN‐γ is widely used in the United States, it is less prescribed in European centers. IFN‐γ was administered to 32 patients with CGD for primary prophylaxis in our study. No beneficial effect was noted in terms of the occurrence of IMI in this group [6, 33, 34, 35]. However, inconsistent access to IFN‐γ in Türkiye, including periods of drug unavailability, may have impacted our cohort's overall effectiveness of prophylaxis.

HSCT is an established curative therapy for CGD, and recent evidence supports its use even in uncontrolled IMI settings [36, 37, 38]. In our study, 13.8% of patients underwent HSCT. We attribute this low HSCT rate to the limited availability of suitable donors and the lack of a specialised BMT centre with sufficient experience.

IMI is the leading cause of mortality in patients with CGD [6, 7]. In our study, 18.0% of patients succumbed to IMI, with an overall IMI‐related mortality rate of 15.2%. Mortality rates varied depending on the diagnostic certainty of the IMI episode, with rates of 33.3% for proven cases, 50% for probable cases, and 20.8% for possible cases. These findings are comparable to those of Blumental et al., who reported a 17% mortality rate associated with invasive fungal infection [29]. The elevated mortality observed in IMI cases underscores the critical need for early diagnosis and effective management in this high‐risk population. The inability to perform biopsies in many cases and the inherent limitations of diagnostic modalities posed challenges. The lack of itraconazole TDM data hindered the evaluation of primary antifungal prophylaxis efficacy.

In conclusion, our study evaluated IMI episodes in a unique cohort with a nearly equal distribution of XL‐CGD and AR‐CGD patients. The findings revealed a persistently high IMI burden (54.1%) among these patients. While itraconazole prophylaxis has demonstrated efficacy in preventing IMI, over one in three patients receiving prophylaxis experienced an IMI episode. The challenges associated with the diagnosis and treatment of these patients need to be addressed to improve outcomes.

## Author Contributions

**Zeynep Ergenc:** conceptualization, investigation, methodology, formal analysis, data curation, writing – original draft, validation, visualization, project administration, writing – review and editing. **Sevgi Bilgic Eltan:** resources, data curation, methodology, investigation, writing – review and editing, writing – original draft, formal analysis. **Betul Gemici Karaaslan:** data curation, resources, methodology, writing – original draft, investigation, visualization. **Ayca Kiykim:** data curation, supervision, resources, writing – review and editing, conceptualization. **Sevgi Aslan Tuncay:** investigation, data curation, conceptualization, visualization. **Seyhan Yilmaz:** investigation, data curation, conceptualization, visualization. **Pinar Canizci Erdemli:** conceptualization, investigation, data curation, visualization. **Aylin Dizi Isik:** data curation, conceptualization, investigation, visualization. **Burcu Parlak:** conceptualization, investigation, data curation, visualization. **Mahir Serbes:** resources, investigation, methodology, project administration, visualization. **Adilia Warris:** writing – review and editing, supervision, methodology, conceptualization, formal analysis, validation. **Ahmet Ozen:** supervision, writing – review and editing, methodology, conceptualization, resources, visualization. **Elif Karakoc‐Aydiner:** conceptualization, methodology, writing – review and editing, supervision, resources, visualization. **Dilek Ozcan:** conceptualization, investigation, data curation, supervision, methodology, visualization, writing – review and editing. **Haluk Cokugras:** supervision, writing – review and editing, conceptualization, visualization, resources, data curation, methodology. **Safa Baris:** conceptualization, supervision, writing – review and editing, methodology, resources, data curation, visualization, writing – original draft. **Eda Kepenekli:** supervision, project administration, writing – review and editing, methodology, conceptualization, writing – original draft, visualization, data curation, validation.

## Conflicts of Interest

A.W. is supported by the Medical Research Council Centre for Medical Mycology (MR/N006364/2) and the NIHR Exeter Biomedical Research Centre (NIHR 203320). The views expressed are those of the author and not necessarily those of the NIHR or the Department of Health and Social Care. Other authors declare no conflicts of interest.

## Data Availability

The data that support the findings of this study are not openly available and available from the corresponding author upon reasonable request.
